# Clinical characteristics that predict parotid abscess: An observational cohort study

**DOI:** 10.1016/j.amsu.2021.102230

**Published:** 2021-03-16

**Authors:** Chonticha Srivanitchapoom, Kedsaraporn Yata

**Affiliations:** Otolaryngology Unit, Phayao Hospital, 269 Moo 11, Tumbon Bantom, Muang Phayao, Phayao, 56000, Thailand

**Keywords:** Parotid abscess, Suppurative parotitis, Parotitis, Deep neck infection, Parotid gland

## Abstract

**Background:**

We analysed clinical factors that are predictive of a diagnosis of parotid abscess among patients with bacterial parotitis.

**Material and methods:**

This retrospective study included 64 hospitalised patients who were diagnosed with parotid abscess, or bacterial parotitis. Data on patient demographics, clinical characteristics, and clinical management were collected. Predictive factors for parotid abscess were evaluated using univariate and multivariate analysis.

**Results:**

There were 25 patients with parotid abscess and 39 with bacterial parotitis. All patients presented with moderate-to-severe disease, required parenteral antibiotics, or had indicators for surgical drainage. Patient profiles and immune status were not significantly associated with parotid abscess. However, parameters that were significantly related to parotid abscess were subacute presentation (approximate 10.4 days) (p value = 0.016), fluctuation (p value < 0.001), and normal (haemoglobin) Hb level >12–13 g/dL (p value = 0.035). Imaging indicated the abscess location, extension and evaluated the complications. Surgical drainage with small skin incision and antibiotic coverage for possible pathogens, in particular *Staphylococcus* spp. and *Streptococcus* spp. produced favourable patient outcomes. Complication was identified in 3 cases with included septicaemia and cellulitis of the face and parapharyngeal space.

**Conclusions:**

Among bacterial parotitis patients, parotid abscess should be considered in whom presented with subacute duration of symptoms, enlarged glands with fluctuation, and non-anaemic problem. Instead of standard skin incision of parotidectomy, small vertical skin incision over a well localised abscess pocket or fluctuated area achieved the good results.

## Highlights

• Parotid abscess should be considered in subacute duration of parotid enlargement.
• There was no related host immunity with parotid abscess formation.
• Favourable treatment outcome achieved from adequate drainage and proper antibiotics.
• Small vertical skin incision provided good results and no re-form an abscess.

## 1 Introduction

Parotitis is defined as inflammation of the parotid glands and may be initiated by several factors including infection [[1], [2], [3], [4], [5]]. Parotitis should be conservatively managed with adequate hydration, good oral hygiene, and antibiotics if indicate [3,6]. However, if treatment is inadequate and/or the disease progresses, suppurative parotitis (SP) or parotid abscess (PA) can develop with their serious complications [2,3,7,8]. A previous study has described the difficulty in distinguishing between PA and parotitis without imaging due to the lack of specific clinical manifestations [9,10]. In addition, treatment of PA requires intervention of pus drainage combines with conservative treatment.

This study sought to identify the clinical factors that are predictive of a diagnosis of PA among patients with bacterial parotitis. We additionally sought to evaluate the clinical characteristics and management strategies of PA and bacterial parotitis.

## 2 Materials and methods

This retrospective cohort study was conducted according to the STROCSS 2019 guidelines [11]. All data were obtained from the otolaryngology unit between May 2015 and December 2019. The study protocol was approved by the IRB of our institute. The study was registered on the Thai Clinical Trails Registry (TCTR 20200619001). Inclusion criteria were as follows: 1) hospitalised patients who were diagnosed with PA or bacterial parotitis; 2) acute or sub-acute duration of disease (within 1 month); and 3) no history of recurrent parotid gland infection. Patients who had a history of parotid gland surgery and associated deep neck infection (DNI) were excluded from this study. PA was defined as a pocket of pus within the parotid parenchyma which was diagnosed by identified the pus collection during the surgical drainage or needle aspiration from the fluctuated area. Bacterial parotitis was defined as symptoms and signs of inflammation over the unilateral parotid gland enlargement with or without the identification of purulent discharge from the orifice of Stensen's duct by spontaneous or gentle massage along the parotid duct. The following patient demographics were recorded: sex, age, body mass index, comorbid diseases, and prior history of medical treatment. Clinical characteristics including presenting symptoms, duration of symptoms, size of abscess pocket, source of parotid infection, and length of hospital stay were also obtained. Clinical examinations included basic laboratory assessment, imaging, and identification of isolated pathogens by aerobic culture, Gram staining and Ziehl–Neelsen staining. Anaerobic bacterial culture was unavailable in our hospital. Drainage techniques, antibiotic dosage and duration, treatment outcomes, and complications were also recorded.

Statistical analyses were conducted using IBM SPSS Statistics for Windows, version 20. All categorical data were reported as a percentage and all continuous data were presented as the mean. The *t*-test was used to compare continuous variables. Clinical data and disease variables were analysed and compared between groups by calculating the odds ratio (OR) and 95% confidence interval (CI). Univariate and multivariate logistic regression analyses were performed to assess the significance of the predisposing factors for parotid abscess. P-values less than 0.05 were considered significant.

## 3 Results

Sixty-four patients met the study criteria, there were 25 patients with PA and 39 with bacterial parotitis. Hospitalisation was indicated in the case of patients who: 1) presented with moderate or severe symptoms that indicated the use of parenteral antibiotics, 2) required surgical drainage, and 3) presented with complications from parotid infection. All clinical characteristics and examination results are described in Table 1 and Table 2.

**Table 1:** Clinical characteristics of hospitalised patients.

	Parotid abscess (%) (n = 25) (39%)	Parotitis (%) (n = 39) (61%)
Sex		
Male	13 (52)	16 (41)
Female	12 (48)	23 (59)
Age in yrs (mean ± SD)	4-90 (52.96 ± 23.34)	7-91 (53.80 ± 20.75)
Age 21–50 (yrs)		
Yes	7 (28)	12 (30.8)
No	18 (72)	27 (69.2)
Age >60 (yrs)		
Yes	10 (40)	16 (41)
No	15 (60)	23 (59)
BMI		
Abnormal	9 (36)	15 (38.5)
Normal	16 (64)	24 (61.5)
Comorbid disease		
Present	14 (56)	21 (53.8)
Absent	11 (44)	18 (46.2)
Cardiovascular disease		
Present	13 (52)	13 (33.3)
Absent	12 (48)	26 (66.7)
Immune disorder		
Present	7 (28)	14 (35.9)
Absent	18 (72)	25 (64.1)
Source of infection		
Present	5 (20)	12 (30.8)
Absent	20 (80)	27 (69.2)
Prior treatment		
Yes	11 (44)	9 (23.1)
No	14 (56)	30 (76.9)
Duration of symptom (d), mean ± SD	3-28 (10.44 ± 7.89)	1-28 (5.92 ± 6.55)
Fluctuation		
Present	10 (40)	–
Absent	15 (60)	39 (100)
BT (^o^C)		
Fever (≥37.5)	8 (32)	9 (23.1)
Normal	17 (68)	30 (76.9)
BT (^o^C)		
High fever (≥38)	3 (12)	4 (10.3)
Normal	22 (88)	35 (89.7)
LOS (d), mean ± SD	2-20 (5.56,3.79)	2-11(4.82 ± 2.17)
Complications		
Present	3 (12)	9 (23.1)
Absent	22 (88)	30 (76.9)

BMI = body mass index; Immune disorder = diabetic mellitus, HIV infection, anaemia, and hematologic disorder post chemoradiation; BT = body temperature; LOS = length of stay.

**Table 2:** Assessment of hospitalised patients.

	Parotid abscess (%) (n = 25) (39%)	Parotitis (%) (n = 39) (61%)
Imaging		
Yes	19 (76)	10 (25.6)
No	6 (24)	29 (74.4)
WBC level		
Abnormal	17 (68)	20 (51.3)
Normal (4800–10000 cells/mm^3^)	8 (32)	19 (48.7)
WBC level >20,000 cells/mm^3^		
Yes	2 (8)	5 (12.8)
No	23 (92)	34 (87.2)
Hb level ≤8 g/dL		
Yes	–	3 (7.7)
No	25 (100)	36 (92.3)
Hb level >10–11 g/dL		
Yes	1 (4)	4 (10.3)
No	24 (96)	35 (89.7)
Hb level >11–12 g/dL		
Yes	5 (20)	10 (25.6)
No	20 (80)	29 (74.4)
Hb level >12–13 g/dL		
Yes	9 (36)	5 (12.8)
No	16 (64)	34 (87.2)
GFR stage		
Stage II-V	10 (40)	17 (43.6)
Stage I	15 (60)	20 (51.3)
N/A	–	2 (5.1)
UA		
Yes	8 (32)	14 (35.9)
Normal	8 (32)	11 (28.2)
UTI	–	3 (7.7)
No	17 (68)	25 (64.1)

WBC = white blood cell count; Hb = haemoglobin; GFR = glomerular filtration rate; N/A = not available; UA = urinary analysis.

### 3.1 Parotid abscess (PA)

PA comprising 39% of inpatient department cases. Male sex was slightly predominant and 56% of patients presented with comorbid disease. Only 7 cases (28%) were defined as immunocompromised, as indicated by a diagnosis of diabetes mellitus (DM), HIV infection, or hematologic problems such as anaemia and/or leukopenia post-chemoradiation for an underlying malignancy. Oro-dental infections that were suspected of being predisposing factors were identified in 5 cases, and 2 cases had an underlying Warthin's tumor of the parotid gland. Eleven cases had been prescribed antibiotics by their primary care physician, 6 of them with first-line antibiotics, while 5 with second-line antibiotics. Common presenting symptoms included afebrile illness, parotid enlargement, and tenderness, with an average duration of 10.4 days. Three patients complained of trismus, one of whom presented with a deep lobe abscess. Parotid gland fluctuation was observed in 10 cases. The majority of abscesses were superficially located (96%) and the average size of the abscess pocket was approximately 3.9 cm. Computed tomography (CT) and ultrasonography (US) scans were used to identify the abscess pocket in 19 cases, while in 6 cases were easy identified by physical examination. The majority of patients showed (haemoglobin) Hb levels were within the normal range. The clinical parameters that significantly distinguished PA from parotitis were a subacute duration of symptoms (p = 0.016, OR(95%CI) = 1.819(0.881–8.153)), fluctuation of the parotid gland (p < 0.001) and normal Hb levels >12–13 g/dL (p = 0.035, OR(95%CI) = 3.835(1.102–13.274)), while multivariate analysis did not reveal any significant parameters. Complications were identified in 3 (12%) patients without any associated significant clinical parameters and included septicaemia and cellulitis of the face and parapharyngeal space (PPS) (Table 3).

**Table 3:** Clinical parameters associated with complications in each group.

Clinical parameter	Parotid abscess complications (n = 3)			Parotitis complications (n = 9)		
	Present	Absent	p -value	Present	Absent	p -value
Immunocompromise host						
Yes	2	5	0.180	1	13	0.081
No	1	17		8	17	
Age ≤ 20 y						
Yes	1	3	0.422	2	1	0.127
No	2	19		7	29	
Age ≥ 60 y						
Yes	–	10	0.198	3	13	0.446
No	3	12		6	17	
Previous treatment						
Yes	1	10	0.593	–	9	0.068
No	2	12		9	21	
BT ≥ 38 °C						
Yes	1	2	0.330	2	2	0.223
No	2	20		7	28	
WBC > 20,000 cells/mm^3^						
Present	–	2	0.770	3	2	0.070
Absent	3	20		6	28	

BT = body temperature; WBC = white blood cell count.

Surgical drainage was performed in 24 cases, while 1 case received a needle aspiration drainage. The preferred approach was a small vertical preauricular incision over the abscess pocket or fluctuated area; however, a parotidectomy involving a standard skin incision was indicated in cases when the abscess was generally confined or located deep within the lobe. There were no post-surgical complications. In most cases, we noted that a thick, white to yellowish, mucoid substance drained from the abscess pocket. We obtained and analysed 24 pus culture specimens, and identified 5 *Staphylococcus* spp., 2 *Klebsiella* spp., 1 *Streptococcus* spp., 1 *Enterobacter* spp., and 1 *Burkholderia* spp., while 14 cases had no growth. Six cases of negative culture received previous antibiotic. Second-line antibiotics (amoxycillin/clavulanic acid or clindamycin) were prescribed for a total treatment course of 10–14 days in the majority of cases, and in selected cases this was combined with 3rd or 4th generation cephalosporin to cover gram-negative bacteria. All patients had regular follow up schedule about 1–2 weeks after completed treatment. An average follow up time was 8.6 days while one melioidosis's patient had 6 months follow up. The treatment outcomes were good, and no disease-related mortality was observed.

### 3.2 Bacterial parotitis

Female sex was dominant, with an average age of 53.8 years (Table 1). The presence of comorbid disease that being classified as immunocompromised was slightly higher in parotitis than PA. In addition, anaemic patients with Hb level ≤8 g/dL had significantly high in parotitis (7.7%). Associated infections that were suspected of being predisposing factors of parotitis were identified in 12 cases (30%) including oro-dental infection, urinary tract infection (UTI), and salivary duct stones. Patients usually presented in acute duration (5.9 day), tender over the affected gland and unilateral gland swelling without fluctuation. Seventeen patients showed purulent discharge from Stensen's duct opening while 22 cases were not identified. Although not statistically significant, the patients usually presented as afebrile, with a slightly increased white blood cell (WBC) count and impaired renal clearance (Table 1, Table 2). Pathogens isolated from the purulent discharge were identified in 4 patients. *Staphylococcus* spp. was identified in 1 case, while 1 case had *Streptococcus* spp., 1 had *Haemophilus* spp. and 1 showed no growth. Imaging by CT scan and US were requested in 10 cases to exclude the possibility of an intra-glandular abscess pocket, and to evaluate the extent and severity of the disease. Complications developed in 9 patients, including 7 with localised cellulitis of the soft tissue surrounding the parotid area, 1 with septicaemia, and 1 with septic shock. In 2 cases with systemic complication, both of them showed pus from parotid duct with *Staphylococcus aureus* was isolated from one patient, other was no growth. Although no significant parameters that associated with complication, immunocompromise host and present with WBC level >20,000 cells/mm^3^ demonstrated of possible predictors (Table 3). Antibiotics selected for use were similar to those used to treat PA with 10–14 days in duration. Conservative treatment and sialagogue produced favourable treatment outcomes. None of the patients required additional surgical drainage. An average follow up time of bacterial parotitis was 10.3 days with good treatment outcomes.

## 4 Discussion

Infectious agents are easily able to infect the parotid gland via 4 routes, including: 1) an intraorally ascending infection via the salivary duct opening; 2) infection of the parenchyma of the primary gland; 3) hematogenous spread; and 4) contiguous spread of peri and/or intra-parotid lymphadenitis into the gland parenchyma following primary site infection [1,3,4,6]. Although the parotid glands are highly susceptible to inflammation and parotitis is a common disorder [12,13], PA and SP are less common and are defined as sequelae of parotitis. They are usually bacterial rather than viral in origin [1,[7], [8], [9],^14^]. PA usually manifest in neonates and premature infants, and are uncommon in adults and children [4,5,9], although some studies have reported that healthy, middle aged individuals are commonly affected [8,10]. This study confirmed that PA can develop in all age groups, and particularly in those who are slightly older (>50 years old). Only 7% of whom had impairment of the immune system, with no specific disease being implicated. These findings were in contrast to those of a previous study of 19,15 and 13 parotid abscess's patients respectively, which reported that immunocompromising conditions were a predisposing factor for parotid abscess [1,4,9]. In addition, normal Hb levels >12–13 g/dL were significantly associated with a diagnosis of PA in our study, thereby excluding anaemia as a predisposing factor. Prior literatures demonstrated predisposing factors for parotitis include oro-dental problems [2], dehydration caused by low fluid intake/living in an arid climate [8], post-operative abdominal surgery [9], a lengthy hospital stay [15], xerostomia from medication [1,16], autoimmune disorders [17], head and neck infection resulting in peri-intra parotid lymphadenitis [4], ductal/parenchymal disorders [1,4,5], specific disseminated diseases such as melioidosis and tuberculosis [18,19], and immunocompromise resulting from diseases such as HIV infection or DM [3,5,9]. In this study, we demonstrated that oro-dental infection was the only predisposing factor of PA while UTI, and salivary duct stones were identified in bacterial parotitis. Although the minority of patient showed specific source of infection, however it can be reflex to the common pathogen, antibiotic selection and also health care prevention.

PA can present as an isolated issue or as a manifestation of other rare diseases [[20], [21], [22]]. Infected Warthin's tumours presenting as parotid abscess have also been reported [13]; 2 of the cases in the present study had this diagnosis. Significant clinical characteristics were periods of subacute symptom presentation of approximately 10.4 days in length. Physical examination usually showed afebrile illness, parotid gland enlargement and pain. Examination findings of trismus can be the result of pain or deep lobe involvement. Fluctuation over the parotid area was specific to abscess formation in this study; however, prior observational studies with high number of patients have described difficulty in identifying fluctuation due to concealment of the abscess pocket by the dense parotid fascia [1]. This study confirmed that imaging by CT scan or US was optimal for the diagnosis of parotid abscess; additionally, these imaging technologies were also used to assess the location and extent of the abscess pocket. Furthermore, imaging was useful in guiding drainage methods and in evaluating complications. However, in cases where complications were suspected, CT scans were preferred over US.

Management strategies included pus drainage and appropriate antibiotic therapy [2,4,[8], [9], [10]]. Second line drugs with amoxycillin/clavulanic acid or clindamycin plus 3rd or 4th generation cephalosporin in selected patients who presented with a high incidence of bacterial gram-negative infections have been previously described [1,2,4,6,9]. Our study additionally recommended a duration of 10–14 days for antibiotic therapy in the most cases. Drainage of the purulent collection within the gland can be performed either by needle aspiration or surgery. The standard skin incision that has been previously reported, using a radical form with modified Blair's incision as parotidectomy or transcervical approach, provides adequate drainage [1,4,23]; however, there is some concern regarding poor wound healing and cosmetic scars associated with this technique [3,8]. A small skin incision over the fluctuated area positioned vertically to the preauricular line has been reported previously with the follow up time of 1–3 weeks [8]. This study also employed a small vertical incision. This incision provided a favourable result, with no cases of re-formation of the abscess, no complications such as facial nerve injury, and no cosmetic deformities. Needle aspiration is an option for diagnosis and drainage and should be performed under guided imaging [3]. However, from our observations, the pus was typically of a thick, mucoid appearance which would be difficult to draw out. A large sized needle under imaging may therefore be indicated. In addition, some authors have expressed concern over the failure of aspiration in a single setting, particularly in rural areas with difficult transportation [2]. *Staphylococcus* spp., anaerobic bacteria and *Streptococcus* spp. are the most commonly reported pathogens [1,6,8,9], and our results were consistent with these findings. *Klebsiella* spp., *Haemophilus* spp., *Enterobacter* spp., *Burkholderia* spp., *Pseudomonas* spp., *Mycobacterium* spp., non-*Mycobacterium* spp., *Propionibacterium* spp., and *Brucella* spp. have also been isolated in previous studies [1,[4], [5], [6],^10^,^16^,^17^,^24^]. Negative bacterial cultures have also been reported in some specimens, due to the use of pre-admission antibiotics [9], or to the presence of other types of pathogenic organisms such as viruses, fungi or parasites [[12], [13], [14]]. Our result also showed 14 cases with negative culture, the possible reasons that indicted negative result might be from 1) received previous antibiotic that may sterilized the pus, 2) no anaerobic culture was obtained and 3) non-suppurative process.

Complication rates associated with PA were low among the inpatient department cases. Only 1 patient had a systemic reaction involving septicaemia, and she received a final diagnosis of melioidosis. No specific clinical parameters were associated with the occurrence of complications. Previous studies have reported a range of complications, including facial nerve palsy [2,6], cervicofacial cellulitis, osteomyelitis of the jaw, temporal lobe abscess [25], DNI (usually involving PPS), craniocervical necrotising fasciitis [26], descending mediastinitis [7], airway obstruction, septicaemia and septic shock [1,5,7]. A persistent salivary fistula may develop after spontaneous rupture of the abscess [2,5]. Additionally, in regions where tuberculosis is endemic, PA caused by *Mycobacterium spp.* have been detected. The clinical manifestations include middle age, chronic parotid enlargement with a median duration of 6 months, a painless mass, facial nerve palsy, and persistent drainage from the gland [19].

Bacterial parotitis is usually causes by *Staphylococcus aureus*, which was isolated in more than 80% of the cases in one study [27], followed by *Streptococcus* spp [28]. and anaerobic bacteria [29]. Our study found similar results of isolated microorganism, although the small number of spontaneous drainage pus were collected. Clinical of bacterial parotitis is diagnosed by the sudden onset of unilateral firm, warm, painful, erythematous swelling of the cheek extending to the angle of the jaw. In addition, patients usually present with toxaemia and marked fever with leucocytosis when the purulent discharge was spontaneous drainage from the orifice of Stensen's duct [29,30]. We observed similar manifestations in this cohort, although we also noted the absence of fluctuated parotid glands. Predisposing factors should be evaluated in all cases, especially UTI which are commonly associated with parotitis. This study suggestion that urinalysis be routinely performed in all cases. In addition, severe anaemia (Hb level ≤8 g/dL) was only associated with bacterial parotitis even though there was not significantly. Prior studies have reported that fever and leucocytosis is also common in acute infection [1,17,28]. In contrast, the patients in this study mostly presented with normal body temperature and slightly increase WBC level.

In some selected bacterial parotitis patients, they required admission especially who were identified the purulent discharge from the duct [31]. Antibiotics should be prescribed for 10–14 days to cover the possible pathogens. In addition, sialagogue and gentle massage over the parotid duct also assisted in achieving favourable treatment outcomes. However, development of the following conditions indicated that re-evaluation with imaging of the patient to exclude parotid abscess 1) lack of clinical improvement in 3–5 days, 2) facial nerve palsy, 3) involve of adjacent structures or deep neck infection, 4) detection of an intraparenchymal abscess formation by clinical examination or radiology, 5) progressive induration or fluctuation or 6) toxicity (septicaemia) [4,29]. Local and systemic reactions such as cellulitis of the surrounding soft tissue and/or septicaemia can complicate bacterial parotitis. Even though, there were not significant clinical predictors associated with complication, the trend of complicated bacterial parotitis cases usually associated with patient with immunocompromise host and WBC >20,000 cells/mm^3^ in this study. In cases of persistence parotid gland enlargement without any signs of inflammation, the following conditions should be excluded: neoplasm, connective tissue disease, alcoholism, gout, uraemia, or sarcoidosis [4,17].

The limitations of this study were as follows: 1) the quality of the data might be affected by the retrospective nature of the study, 2) there was not sufficient evidence to support the diagnosis of bacterial parotitis due to the limitations of viral testing in our laboratory, 3) only selected parotitis patients received imaging that definitely excluded parotid abscess, and 4) small number of PA patients.

## 5 Conclusion

Among parotitis patients, PA should be considered in whom presented with subacute duration of symptoms, enlarged glands with fluctuation, and non-anaemic problem. Patient with PA should be considered for hospital admission for intravenous antibiotic and surgical pus drainage. Imaging should be performed in all cases to confirm the diagnosis, assess the location of the abscess pocket, and evaluate the complications. Instead of standard skin incision of parotidectomy, small vertical skin incision over the fluctuated or well-defined abscess pocket achieved good results and the abscess did not re-form. This approach minimised issues with wound healing and cosmetic scarring.

## Ethical approval

The study protocol was approved by the IRB of our institute. Study code 63-02-002.

## Source of funding

None.

## Author contribution

(1) the conception and design of the study, or acquisition of data, or analysis and interpretation of data, (2) drafting the article or revising it critically for important intellectual content, (3) final approval of the version to be submitted.

## Guarantor

Chonticha Srivanitchapoom,M.D.

## Consent

This study not related to consent form.

## Registration of research studies

1. Name of the registry: Thai Clinical Trials Registry.

2. Unique Identifying number or registration ID: TCTR20200619001.

3. Hyperlink to your specific registration (must be publicly accessible and will be checked): http://www.thaiclinicaltrials.org/=> then search for “Clinical characteristics that predict parotid abscess: an observational study”.

## Declarations of interest

None.

## Disclosure

The authors have nothing disclosure.

## Data statement

The research data is confidential.

## Provenance and peer review

Not commissioned, externally peer-reviewed.

## Declaration of competing interest

All authors have no conflicts of interest.
